# The H240R Protein of African Swine Fever Virus Inhibits Interleukin 1β Production by Inhibiting NEMO Expression and NLRP3 Oligomerization

**DOI:** 10.1128/jvi.00954-22

**Published:** 2022-11-03

**Authors:** Pingping Zhou, Jingwen Dai, Kehui Zhang, Tao Wang, Lian-Feng Li, Yuzi Luo, Yuan Sun, Hua-Ji Qiu, Su Li

**Affiliations:** a State Key Laboratory of Veterinary Biotechnology, National African Swine Fever Para-reference Laboratory, National High Containment Facilities for Animal Diseases Control and Prevention, Harbin Veterinary Research Institute, Chinese Academy of Agricultural Sciences, Harbin, China; b Northeast Agricultural University, Harbin, China; c Harbin Medical University, Harbin, China; Hudson Institute of Medical Research

**Keywords:** African swine fever virus, H240R protein, NF-κB, NLRP3 inflammasome, interleukin 1β

## Abstract

The H240R protein (pH240R), encoded by the *H240R* gene of African swine fever virus (ASFV), is a 241-amino-acid capsid protein. We previously showed that the deletion of *H240R* from the ASFV genome, creating ASFV-ΔH240R, resulted in an approximately 2-log decrease in infectious virus production compared with the wild-type ASFV strain (ASFV-WT), and ASFV-ΔH240R induced higher interleukin 1β (IL-1β) production in porcine alveolar macrophages (PAMs) than did ASFV-WT, but the underlying mechanism remains to be elucidated. Here, we demonstrate that the activation of the NF-κB signaling and NLRP3 inflammasome was markedly induced in PAMs upon ASFV-ΔH240R infection compared with ASFV-WT. Moreover, pH240R inhibited NF-κB activation by interacting with NEMO and promoting the autophagy-mediated lysosomal degradation of NEMO, resulting in reduced pro-IL-1β transcription. Strikingly, NLRP3 deficiency in PAMs inhibited the ASFV-ΔH240R-induced IL-1β secretion and caspase 1 activation, indicating an essential role of NLRP3 inflammasome activation during ASFV-ΔH240R replication. Mechanistically, pH240R interacted with NLRP3 to inhibit its oligomerization, leading to decreased IL-1β production. Furthermore, the inhibition of the NF-κB signaling and NLRP3 inflammasome activation promoted ASFV-ΔH240R replication in PAMs. Taken together, the results of this study reveal an antagonistic mechanism by which pH240R suppresses the host immune response by manipulating activation of the NF-κB signaling and NLRP3 inflammasome, which might guide the rational design of live attenuated vaccines or therapeutic strategies against ASF in the future.

**IMPORTANCE** African swine fever (ASF), a lethal hemorrhagic disease, is caused by African swine fever virus (ASFV). There are no commercially available vaccines or antivirals for the disease. Here, we showed that ASFV with a deletion of the *H240R* gene exhibits high-level expression of interleukin 1β (IL-1β), a proinflammatory cytokine, in porcine alveolar macrophages and that the H240R protein (pH240R) exhibits robust inhibitory effects on IL-1β transcription and production. More specifically, pH240R inhibited NF-κB activation via the autophagy-mediated lysosomal degradation of NEMO, leading to the decrease of pro-IL-1β transcription. In addition, pH240R interacted with NLRP3 to inhibit its oligomerization, leading to decreased IL-1β production. Our results indicate that pH240R is involved in the evasion of host innate immunity and provide a novel target for the development of a live attenuated vaccine against ASF.

## INTRODUCTION

African swine fever (ASF), a lethal hemorrhagic disease, is caused by African swine fever virus (ASFV); there are no commercially available vaccines or antiviral strategies for this disease (1–3). ASFV is a member of the family *Asfarviridae* and the only known DNA arbovirus. ASFV particles are 260 to 300 nm in diameter and composed of complex multilayered structures with an overall icosahedral morphology (4, 5). ASFV is composed of five distinct structures, namely, an outer icosahedral protein capsid containing an inner lipid envelope, a core shell, a double-stranded DNA genome, and an external lipid envelope. The viral genome is a double-stranded DNA that is 170 to 194 kb in length and encodes more than 150 viral proteins, which are involved in viral replication, virus-host interactions, and modulation of the host innate immune responses (6). A previous study identified 68 different structural proteins in the virion by mass spectrometry analysis (7), and these proteins included multiple components associated with the virus structure as well as a set of enzymes involved in viral mRNA transcription or modification and the maintenance of viral genome integrity. Although the functions of several ASFV genes have been elucidated, large gaps remain in the knowledge about the functions of viral structural proteins, which hinders vaccine and antiviral strategy development.

*H240R* encodes a structural protein that is relatively abundant in the virion (7). Our previous study showed that the H240R protein (pH240R) is a capsid protein, and it is not absolutely essential for virus replication *in vitro*. However, the infectious-virus titers from porcine alveolar macrophages (PAMs) infected with a recombinant ASFV with *H240R* deleted (ASFV-ΔH240R) are approximately 100-fold lower than those from the cells with the wild-type virus (ASFV-WT) (8). Unexpectedly, higher transcription and production of the proinflammatory cytokine interleukin 1β (IL-1β) were observed in PAMs infected with ASFV-ΔH240R than ASFV-WT (8).

IL-1β, which is primarily produced by macrophages, is a key mediator of innate immune responses to various invading pathogens (9, 10), such as adenovirus (11), Zika virus (12) and respiratory syncytial virus (13). Importantly, the production of biologically active IL-1β is mediated by two distinct innate immune signaling cascades. The first signaling cascade involves transcriptional activation of nuclear factor κB (NF-κB), leading to the synthesis of mRNAs that encode pattern recognition receptors (PRRs) (14–16). The second signaling cascade includes PRR-induced assembly of a multiprotein complex called the inflammasome and the maturation of caspase 1, which leads to the cleavage of pro-IL-1β into the mature IL-1β (17). Secretion of IL-1β from cells infected with pathogens is closely regulated by inflammasomes. An inflammasome typically consists of a sensor protein, the adaptor molecule apoptosis-associated speck-like molecule containing a caspase recruitment domain (ASC), and the zymogen procaspase 1 (18, 19). Assembly of the inflammasome is triggered by the recognition of a variety of stimuli by sensor proteins. The activated sensor protein recruits procaspase 1 by ASC, leading to the autocleavage of caspase 1 to generate an active caspase 1-p10/p20 tetramer. Then, the activated caspase 1 cleaves inactive pro-IL-1β, resulting in IL-1β production. Ultimately, the cytokines processed by the inflammasome contribute to the antiviral response by establishing a proinflammatory state in tissues, which in turn facilitates the recruitment and activation of immune cells (20, 21), the establishment of a cellular antiviral state (22), and the induction of pyroptotic cell death (23).

IL-1β plays an important role in the control of infection by various pathogens, as described above. However, pathogens antagonize the host antiviral responses to replicate and exert pathogenic effects *in vivo* (9, 10). These observations suggest that pathogens have evolved several strategies to evade the protective innate immune responses regulated by proinflammatory cytokines. Previous studies have shown that several viruses counteract NF-κB and inflammasome activation (12, 24–30). However, the correlation between the NF-κB signaling and/or inflammasome activation and viral replication remains unclear *in vitro*.

ASFV infection can induce low-level IL-1β and tumor necrosis factor alpha (TNF-α) in pigs as early as 7 days postinfection (31). The ASFV L83L protein specifically interacts with IL-1β, and the ASFV mutant with *L83L* deleted is not attenuated in pigs (32). pA238L inhibits the expression of IL-1β by inhibiting the activation of NF-κB, and the mutant ASFV with *A238L* deleted remains virulent in pigs (33). The ASFV pMGF505-7R specifically interacts with the IKK complex to inhibit pro-IL-1β transcription, and a mutant ASFV with *MGF505-7R* deleted exhibits attenuated phenotype in pigs and induces high-level IL-1β *in vitro* and *in vivo* (34). We have recently shown that pH240R is involved in the ASFV replication cycle, and the H240R-deleted ASFV induced greater IL-1β response than did ASFV-WT in PAMs. Moreover, it has not been elucidated that the relationship between the phenotype with reduced ASFV-ΔH240R replication and IL-1β production in ASFV-ΔH240R infection. We speculate that pH240R may inhibit the NF-κB signaling and/or inflammasome activation to regulate downstream inflammatory responses. Therefore, we investigated the effects of pH240R on IL-1β production in PAMs during viral infection.

Here, we demonstrated that the NF-κB signaling was activated in the ASFV-ΔH240R-infected PAMs by measuring the transcription of *pro-IL-1β* and the phosphorylation of IκBα and p65. Mechanistically, pH240R interacted with the adaptor protein NEMO of the IKK complex. Importantly, pH240R inhibited NF-κB activation by degrading NEMO, leading to the decrease of *pro-IL-1β* transcription. Intriguingly, ASFV-ΔH240R infection induced NLRP3 inflammasome activation to promote IL-1β secretion from PAMs. Moreover, pH240R interacted with NLRP3 to inhibit its oligomerization, leading to decreased IL-1β production. Altogether, our study reveals that pH240R counteracts the first and second signaling pathways of IL-1β production, highlighting the importance of IL-1β in the host antiviral immune responses against ASFV infection.

## RESULTS

### The *H240R* gene-deleted ASFV activates the NF-κB signaling pathway in PAMs.

**FIG 1:**
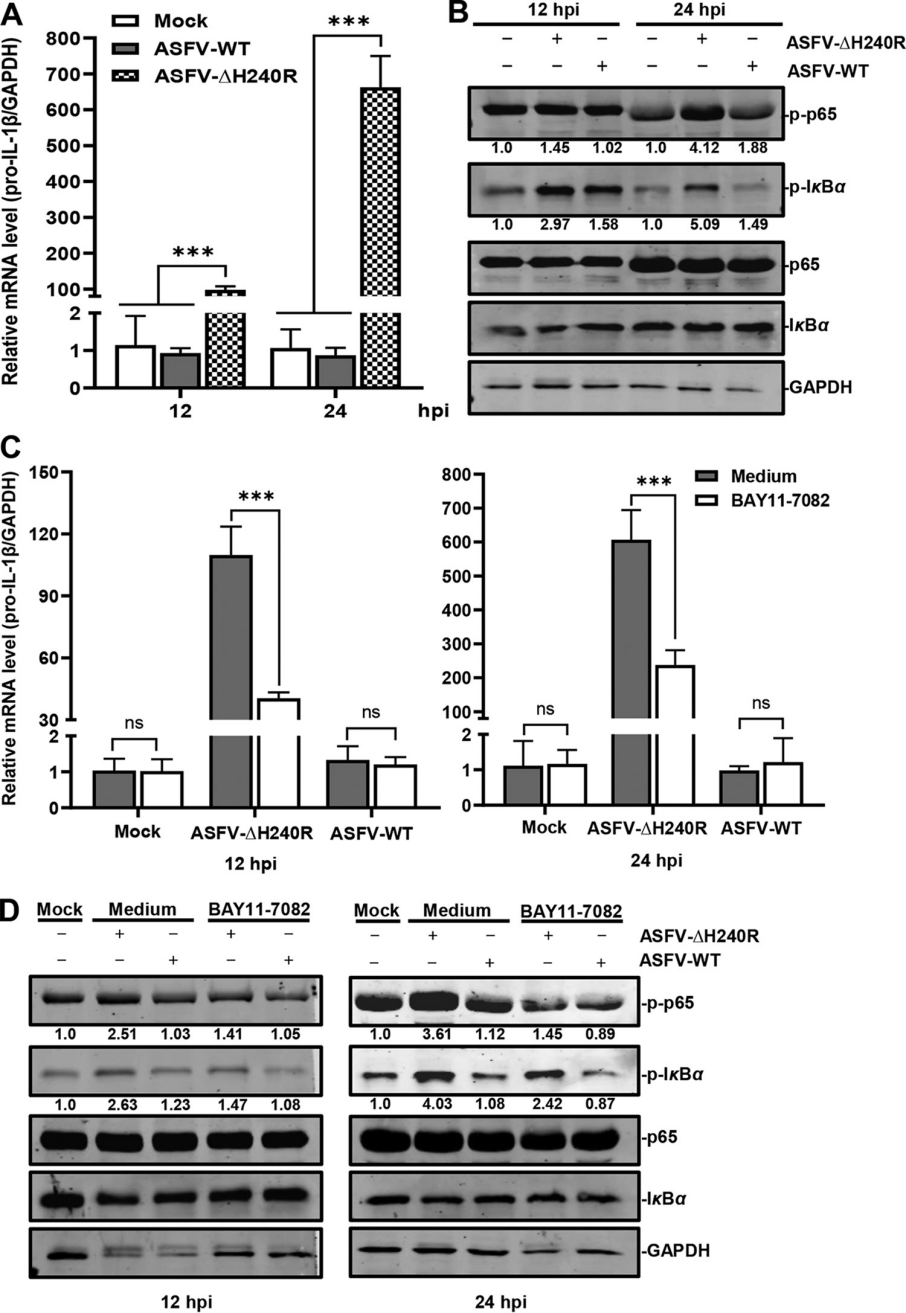
ASFV-ΔH240R infection activates the NF-κB signaling to promote *pro-IL-1β* transcription. (A) *pro-IL-1β* transcription was upregulated in PAMs infected with ASFV-ΔH240R compared with ASFV-WT. PAMs were infected with equal genome copies of ASFV-ΔH240R and ASFV-WT for 12 h or 24 h, and *pro-IL-1β* transcription was examined by RT-qPCR using primers against *pro-IL-1β* and *GAPDH*. (B) The phosphorylation levels of IκBα and p65 were increased in the PAMs infected with ASFV-ΔH240R compared with ASFV-WT. PAMs were infected with equal genome copies of ASFV-ΔH240R and ASFV-WT for 12 or 24 h, and the phosphorylation levels of IκBα and p65 were analyzed by Western blotting. The protein band intensities were analyzed by Image Studio software. (C) *pro-IL-1β* transcription was decreased in the inhibitor-treated PAMs induced by ASFV-ΔH240R infection. BAY11-7082, an inhibitor of the NF-κB signaling pathway, was added to the PAMs infected with ASFV-ΔH240R or ASFV-WT, and the transcription of *pro-IL-1β* was analyzed by RT-qPCR at 12 or 24 hpi. (D) The phosphorylation levels of IκBα and p65 were decreased in the inhibitor-treated PAMs induced by ASFV-ΔH240R infection. BAY11-7082 was added to the PAMs infected with ASFV-ΔH240R or ASFV-WT, and the phosphorylation levels of IκBα and p65 were analyzed by Western blotting at 12 or 24 hpi. Data are from three independent experiments. The error bars denote standard errors of the means. The results of densitometry analysis to quantify the ratio of p-p65 or p-IκBα to GAPDH are shown at the bottom. The significance of differences between groups (*n *= 3) was determined using Student’s *t* test (***, *P < *0.001; ns, not significant).

IκBα acts as a regulator of the NF-κB signaling pathway, and the phosphorylation of IκBα and p65 is the indicator of NF-κB activation (35). To determine whether the NF-κB signaling pathway is activated in the ASFV-ΔH240R-infected PAMs, the transcription of *pro-IL-1β* was analyzed by using reverse transcription-quantitative PCR (RT-qPCR), and the phosphorylation levels of IκBα and p65 were measured by using Western blotting at 12 and 24 h postinfection (hpi). The results showed that the transcription levels of *pro-IL-1β* were increased by approximately 100- and 600-fold at 12 and 24 hpi, respectively, in the PAMs infected with ASFV-ΔH240R compared with those with ASFV-WT (Fig. 1A). The phosphorylation levels of IκBα and p65 in ASFV-ΔH240R-infected PAMs were increased by 1.88- and 1.45-fold at 12 h (Fig. 1B, left) and by 3.42- and 2.20-fold at 24 h (Fig. 1B, right) compared with those in the ASFV-WT-infected cells. These results indicate that the NF-κB signaling is activated upon ASFV-ΔH240R infection.

To determine if the NF-κB signaling pathway is specifically activated by ASFV-ΔH240R infection, BAY11-7082, an inhibitor of the NF-κB signaling pathway, was used to treat PAMs, and the transcription levels of *pro-IL-1β* and the phosphorylation levels of IκBα and p65 were analyzed at 12 and 24 hpi as described above. The results showed that the transcription levels of *pro-IL-1β* in the BAY11-7082-treated PAMs were decreased by 2.75- and 2.65-fold at 12 and 24 h, respectively, compared with those in the medium-treated PAMs upon ASFV-ΔH240R infection (Fig. 1C). The phosphorylation levels of IκBα and p65 in the inhibitor-treated PAMs were decreased by 1.78- and 1.67-fold at 12 h and by 2.66- and 2.48-fold at 24 h, compared with those in the medium-treated PAMs with ASFV-ΔH240R infection (Fig. 1D). However, there were no significant differences in the transcription of *pro-IL-1β* and the phosphorylation levels of IκBα and p65 between the inhibitor- and the medium-treated PAMs infected with ASFV-WT. The data suggest that the activation of NF-κB induced by ASFV-ΔH240R infection is suppressed by the inhibitor of the NF-κB pathway.

Taken together, our results demonstrate that ASFV-ΔH240R infection specifically activates the NF-κB signaling to facilitate *pro-IL-1β* transcription.

### pH240R inhibits the activation of the NF-κB signaling pathway.

**FIG 2:**
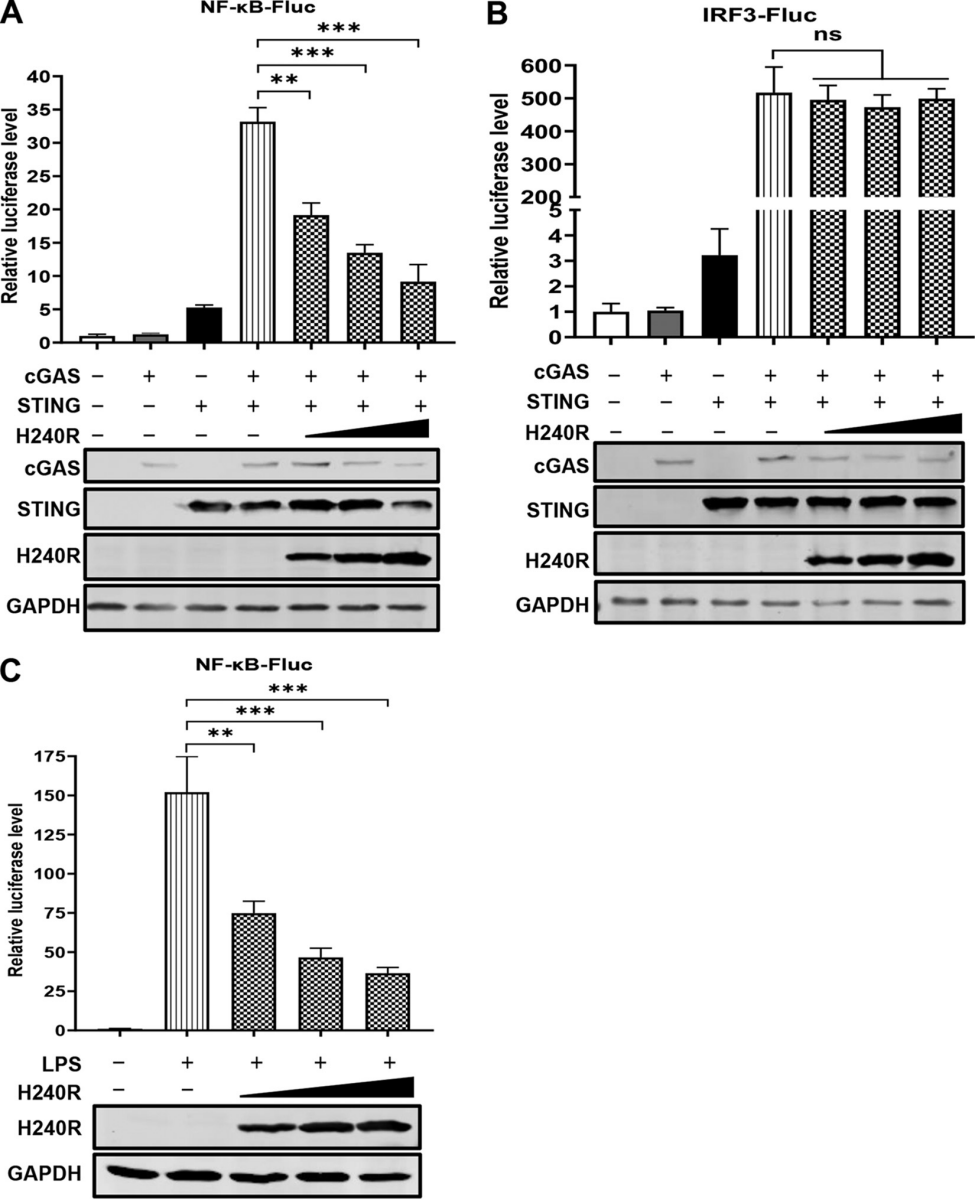
The H240R protein (pH240R) inhibits the activation of the NF-κB signaling induced by cGAS-STING or LPS stimulation. (A and B) The effects of pH240R on the activation of the NF-κB (A) and IRF3 promoters (B) induced by overexpression of cGAS and STING. HEK293T cells were cotransfected with pFlag-cGAS, pFlag-STING, pNF-κB-FLuc, or pIRF3-FLuc (0.1 μg) and pTK-RLuc (0.01 μg) in combination with pCAGGS-Flag or different amounts of pFlag-H240R, and the luciferase activity was measured at 24 hpt. The expression of each protein was measured by Western blotting. (C) pH240R inhibited the NF-κB promoter activation induced by LPS stimulation. HEK293T cells were cotransfected with pCAGGS-Flag and different amounts of pFlag-H240R for 24 h, followed by treatment with or without LPS (100 μM) for another 12 h, and the luciferase activity was determined. The expression of pH240R and GAPDH were measured by Western blotting. Data are from three independent experiments. The error bars denote standard errors of the means. The significance of the difference between groups (*n *= 3) was determined using Student’s *t* test (**, *P < *0.01; ***, *P < *0.001; ns, not significant).

The transcription and expression of pro-IL-1β are regulated by the NF-κB signaling (36). To investigate the effects of pH240R on the NF-κB promoter activation, HEK293T cells were cotransfected with the NF-κB reporter and different amounts of the *H240R*-expressing plasmid, in combination with *cGAS*- and *STING*-expressing plasmids or stimulation with lipopolysaccharides (LPS), and then the cells were lysed to examine the luciferase signals. As shown in Fig. 2A and B, ectopic expression of pH240R inhibited the cGAS/STING-induced activation of the NF-κB reporter but not the IRF3 reporter in a dose-dependent manner. In addition, pH240R inhibited the activation of the NF-κB reporter in the LPS-stimulated HEK293T cells compared with the mock-treated HEK293T cells (Fig. 2C). These results suggest that pH240R efficiently inhibits the activation of the NF-κB signaling.

### pH240R inhibits proinflammatory cytokine expression.

**FIG 3:**
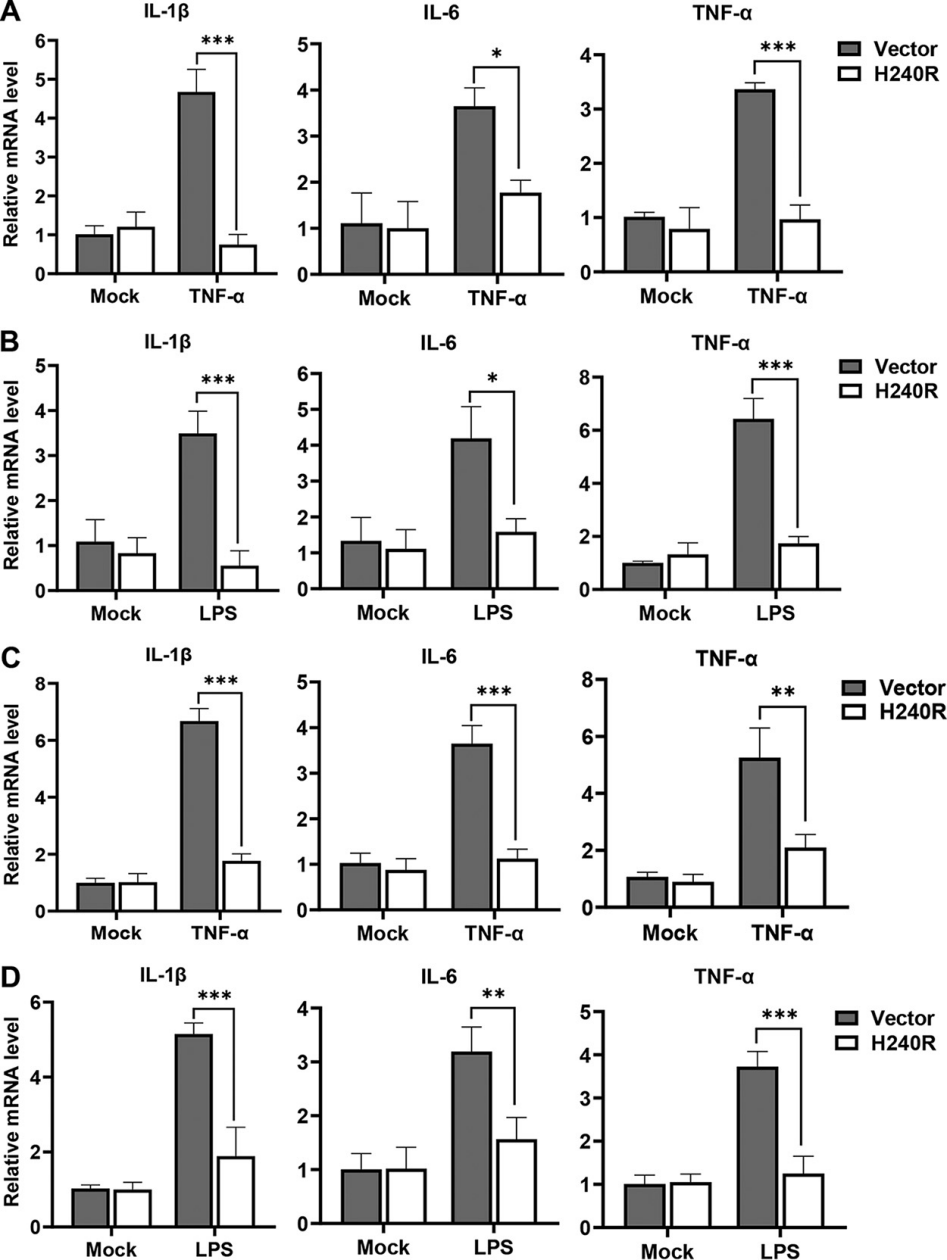
The H240R protein (pH240R) inhibits TNF-α- or LPS-induced proinflammatory cytokine expression. (A and B) pH240R inhibited TNF-α- or LPS-induced proinflammatory cytokine expression. HEK293T cells were transfected with pCAGGS-Flag or pFlag-H240R for 24 h, followed by treatment with or without TNF-α (100 μg/mL) (A) or LPS (100 μM) (B) for another 10 h, and the transcription levels of *IL-1β*, *IL-6*, and *TNF-α* were measured by RT-qPCR. (C and D) pH240R inhibited TNF-α- or LPS-induced proinflammatory cytokine expression. PAMs were transfected with pCAGGS-Flag or pFlag-H240R for 24 h, followed by treatment with or without TNF-α (100 μg/mL) (A) or LPS (100 μM) (B) for another 10 h, and the transcription levels of *IL-1β*, *IL-6*, and *TNF-α* were measured by RT-qPCR. Data are from three independent experiments. The error bars denote standard errors of the means. The significance of the difference between groups (*n *= 3) was determined using Student’s *t* test (*, *P < *0.05; **, *P < *0.01; ***, *P < *0.001).

TNF-α- or LPS-induced NF-κB activation facilitates the expression of various proinflammatory cytokines, including IL-1β, IL-6, and TNF-α (37). We have shown that pH240R inhibited the activation of the NF-κB signaling; thus, we examined whether pH240R inhibited the expression of these proinflammatory cytokines. The effects of pH240R on the TNF-α- and LPS-induced transcription of *IL-1β*, *IL-6*, and *TNF-*α were evaluated in HEK293T cells and PAMs. Stimulation with TNF-α efficiently induced the transcription of IL-1β, IL-6, and TNF-α, while overexpression of pH240R significantly inhibited the TNF-α-induced expression of these proinflammatory cytokines (Fig. 3A). As expected, LPS stimulation efficiently induced the expression of IL-1β, IL-6, and TNF-α, and overexpression of pH240R significantly inhibited the LPS-induced expression of these proinflammatory cytokines (Fig. 3B). Similarly, pH240R significantly suppressed the expression of proinflammatory cytokines in PAMs (Fig. 3C and D). These data suggest that pH240R significantly inhibits the expression of proinflammatory cytokines.

### pH240R interacts with the NEMO of the IKK complex.

**FIG 4:**
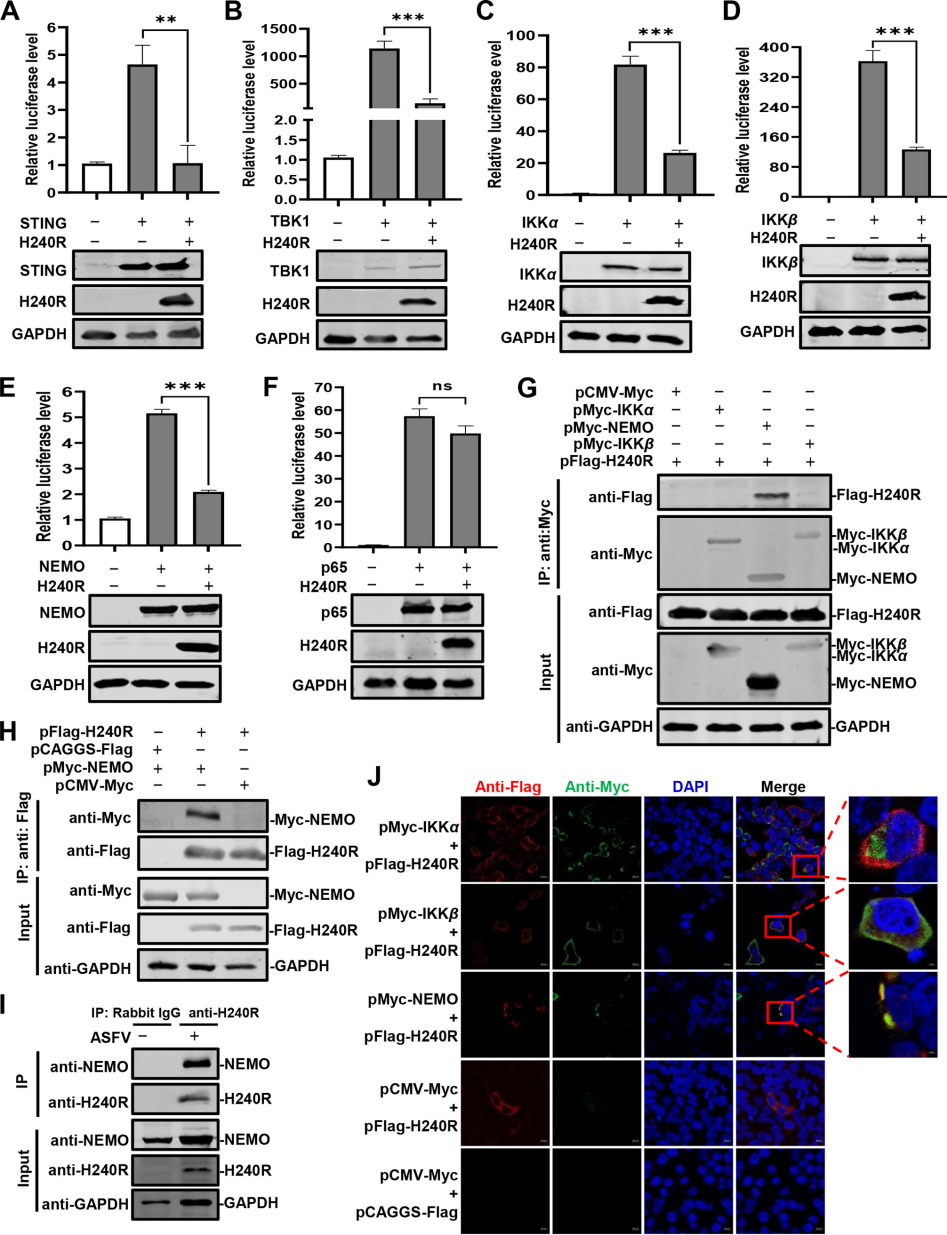
The H240R protein (pH240R) interacts with the NEMO of the IKK complex. (A and F) pH240R inhibited STING-, TBK1-, IKKα-, IKKβ-, and NEMO-induced but not p65-induced NF-κB promoter activation. HEK293T cells were cotransfected with pNF-κB-FLuc (0.1 μg) and pTK-RLuc (0.01 μg), in combination with pFlag-STING (A), pFlag-TBK1 (B), pMyc-IKKα (C), pMyc-IKKβ (D), pMyc-NEMO (E), or pMyc-p65 (F), and pCAGGS-Flag or pFlag-H240R for 24 h. Then, the luciferase activity was measured by a double luciferase report assay system, and the expression of each protein was examined by Western blotting using an anti-Flag or anti-Myc MAb. (G and H) pH240R interacted with NEMO. HEK293T cells were cotransfected with pCMV-Myc, pMyc-IKKα, pMyc-IKKβ, or pMyc-NEMO in combination with pFlag-H240R. At 48 hpt, the cell lysates were subjected to a co-IP assay with the anti-Myc MAb (G) and the anti-Flag MAb (H) and analyzed with the indicated antibodies by Western blotting. (I) Endogenous co-IP assay. PAMs were infected with ASFV (multiplicity of infection [MOI] = 3) for 48 h and subjected to a co-IP assay using anti-H240R PAb, and irrelevant rabbit IgG was used as a negative control. (J) Colocalization of pH240R with NEMO. HEK293T cells were transfected with pCMV-Myc or pFlag-H240R alone and cotransfected with pMyc-IKKα, pMyc-IKKβ, or pMyc-NEMO in combination with pFlag-H240R. IKKα, IKKβ, NEMO, and pH240R and were then analyzed by laser confocal microscopy. Bars, 10 μm. Data are from three independent experiments. The error bars denote standard errors of the means. The significance of the difference between groups (*n *= 3) was determined using Student’s *t* test (**, *P < *0.01; ***, *P < *0.001; ns, not significant).

To further investigate the mechanisms underlying the pH240R-mediated inhibition of NF-κB activation, we first determined whether pH240R is associated with various adaptor proteins (STING, TBK1, IKKα, IKKβ, or NEMO) that are involved in the activation of the NF-κB promoter. The IKK complex is composed of IKKα, IKKβ, and the regulatory ubiquitin-binding protein NEMO, which regulates the activity of the kinase subunits IKKα and IKKβ. The data showed that pH240R markedly inhibited the activation of the NF-κB promoter induced by STING, TBK1, IKKα, IKKβ, or NEMO (Fig. 4A to E) but not by p65 (Fig. 4F), suggesting that pH240R might negatively regulate NF-κB activation by targeting the IKK complex.

To identify the adaptor proteins that interact with pH240R to facilitate its inhibition of NF-κB activation, cGAS, STING, TBK1, IKKα, IKKβ, and NEMO were selected, and the interactions of these proteins with pH240R were validated by coimmunoprecipitation (co-IP) assay. The results showed that the Flag-tagged cGAS, STING, or TBK1 was not coprecipitated with the Myc-tagged pH240R (data not shown), while the Myc-tagged NEMO, but not the Myc-tagged IKKα or IKKβ, was coprecipitated with the Flag-tagged pH240R (Fig. 4G). The Flag-tagged pH240R was coprecipitated with the Myc-tagged NEMO upon incubation with anti-Flag monoclonal antibody (MAb) and protein G-agarose (Fig. 4H). To determine whether pH240R interacts with the NEMO in the context of ASFV infection, virus-infected cell lysates were immunoprecipitated with rabbit anti-pH240R polyclonal antibody (PAb) and probed for the presence of NEMO. As a result, NEMO could be detected in the ASFV-infected cells (Fig. 4I). We also investigated whether pH240R colocalizes with NEMO in HEK293T cells cotransfected with the *H240R*- and *NEMO*-expressing plasmids. Consistent with the above results, the colocalization of pH240R with NEMO, but not with IKKα or IKKβ, was observed in the cytoplasm of HEK293T cells with a Pearson’s correlation coefficient of 0.93 by laser confocal microscopy (Fig. 4J). These data suggest that pH240R inhibits NF-κB activation by interacting with NEMO.

### pH240R antagonizes NF-κB activation by promoting the autophagy-mediated lysosomal degradation of NEMO.

**FIG 5:**
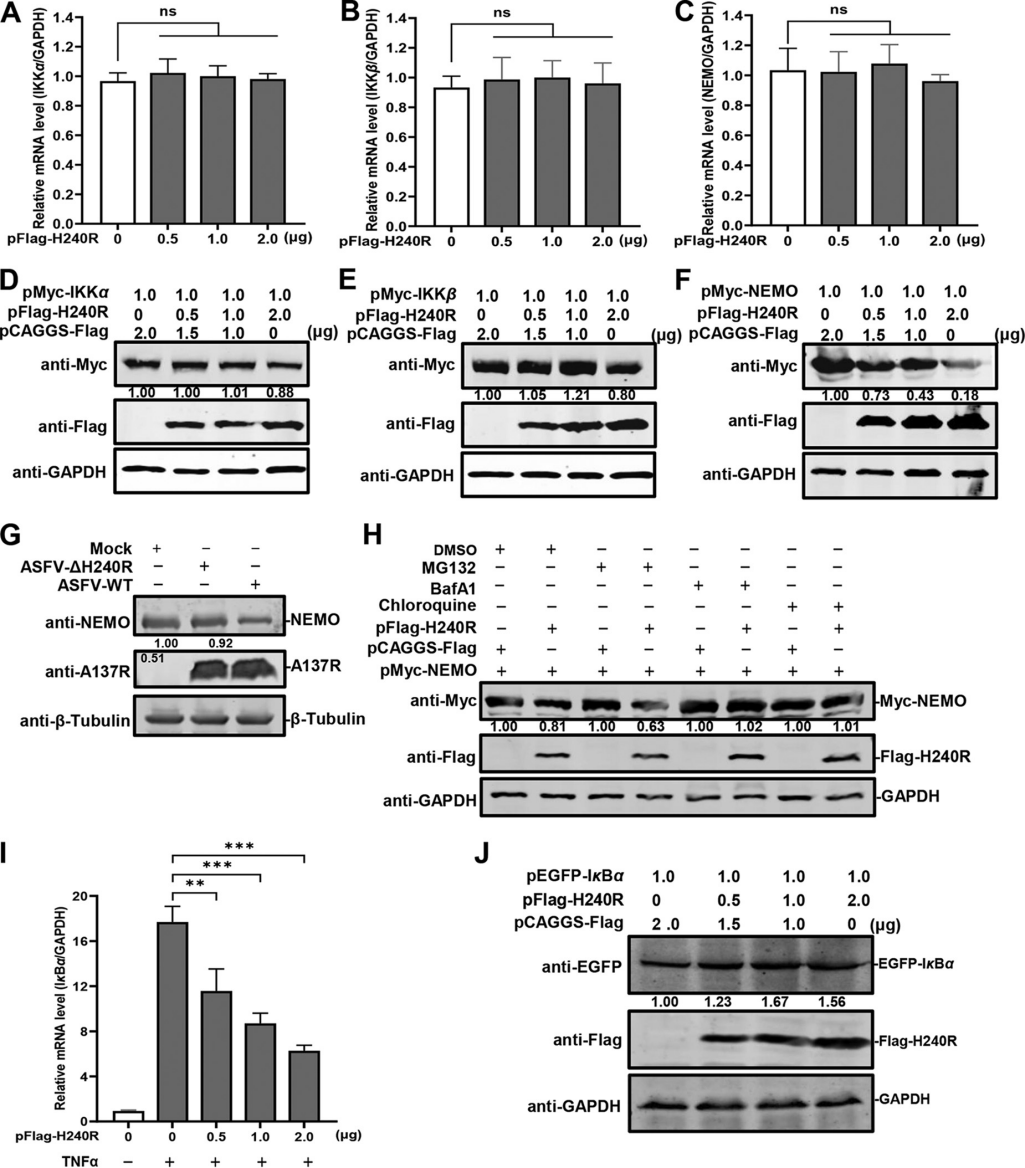
The H240R protein (pH240R) enhances the autophagy-mediated lysosomal degradation of NEMO. (A to C) The transcription levels of *IKKα*, *IKKβ*, and *NEMO* were not affected by pH240R. HEK293T cells were cotransfected with pMyc-IKKα (A), pMyc-IKKβ (B), and pMyc-NEMO (1 μg) (C) with increasing doses of pFlag-H240R (0, 0.5, 1.0, and 2.0 μg) into 12-well plates, and the transcription levels of *IKKα*, *IKKβ*, and *NEMO* were detected by RT-qPCR. (D to F) pH240R inhibited NEMO expression. HEK293T cells were cotransfected with pMyc-IKKα, pMyc-IKKβ, pMyc-NEMO (1 μg) and increasing doses of pFlag-H240R (0, 0.5, 1.0, and 2.0 μg) into 12-well plates. The expression of IKKα (D), IKKβ (E), and NEMO (F) were examined by Western blotting. (G) ASFV-WT infection induces NEMO downregulation. PAMs were infected with ASFV or ASFV-ΔH240R (MOI = 3) for 48 h, and the expression of each protein was determined by Western blotting. (H) pH240R promotes the autophagy-mediated lysosomal degradation of NEMO. pMyc-NEMO was transfected with or without pFlag-H240R into HEK293T cells and then treated with dimethyl sulfoxide (DMSO), MG132 (10 μM), chloroquine (20 mM), or bafilomycin A1 (20 mM) for 18 h. (I) The transcription of endogenous IκBα is downregulated by pH240R. HEK293T cells were transfected with the increasing doses of pFlag-H240R (0, 0.5, 1.0, and 2.0 μg) into 24-well plates for 24 h, and then stimulated with TNF-α for an additional 8 h. The transcription of IκBα was determined by RT-qPCR. (J) pH240R stabilizes the IκBα expression. HEK293T cells were cotransfected with pEGFP-IκBα (1 μg) and increasing doses of pFlag-H240R (0, 0.5, 1.0, and 2.0 μg) into 12-well plates. The expression of IκBα was detected by Western blotting. The results of densitometry analysis to quantify the ratio of Myc-IKKα, Myc-IKKβ, Myc-NEMO, or EGFP-IκBα to GAPDH are shown at the bottom. **, *P < *0.01; ***, *P < *0.001; ns, not significant.

To investigate the effects of pH240R on the expression of NEMO, which is a polyubiquitin-binding protein (38), pMyc-IKKα, pMyc-IKKβ, or pMyc-NEMO and increasing amounts of pFlag-H240R were cotransfected into HEK293T cells. The results showed that pH240R did not affect the transcription of *IKK*α, *IKKβ*, or *NEMO* (Fig. 5A to C). However, pH240R reduced the expression of NEMO, but not that of IKKα or IKKβ, in a dose-dependent manner (Fig. 5D to F). The expression of NEMO was lower in the ASFV-infected PAMs than that in the ASFV-ΔH240R-infected PAMs (Fig. 5G). There are two main pathways for protein degradation, i.e., the proteasome pathway and lysosomal proteolysis. To determine the pathways involved in the NEMO degradation by pH240R, the proteasome inhibitor MG132 and the lysosome inhibitors chloroquine and bafilomycin A1 (BafA1) were used to treat the cells. The results showed that the pH240R-induced NEMO degradation was rescued by chloroquine or BafA1 but not by MG132 (Fig. 5H), indicating that pH240R downregulates NEMO levels via autophagy-mediated lysosomal degradation. IκBα is a regulator of NF-κB activation and binds tightly to NF-κB dimers (39). Phosphorylation by the IKK complex triggers IκBα ubiquitination and degradation. To investigate whether pH240R functions as an inhibitor of the degradation of IκBα, the increasing amounts of pFlag-H240R were transfected into HEK293T cells for 24 h, and then stimulated with TNF-α to analyze the transcription levels of endogenous IκBα. The results showed that pH240R reduced the transcription of endogenous IκBα in a dose-dependent manner (Fig. 5I). pEGFP-IκBα and increasing amounts of the *H240R*-expressing plasmid were cotransfected into HEK293T cells, and the expression of IκBα was analyzed. The results showed that pH240R stabilized the expression of IκBα in a dose-dependent manner (Fig. 5J).

Taken together, our results demonstrate that pH240R inhibits NF-κB activation by degrading NEMO, leading to the decrease of *pro-IL-1β* transcription.

### ASFV-ΔH240R activates the NLRP3 inflammasome to induce IL-1β secretion in PAMs.

**FIG 6:**
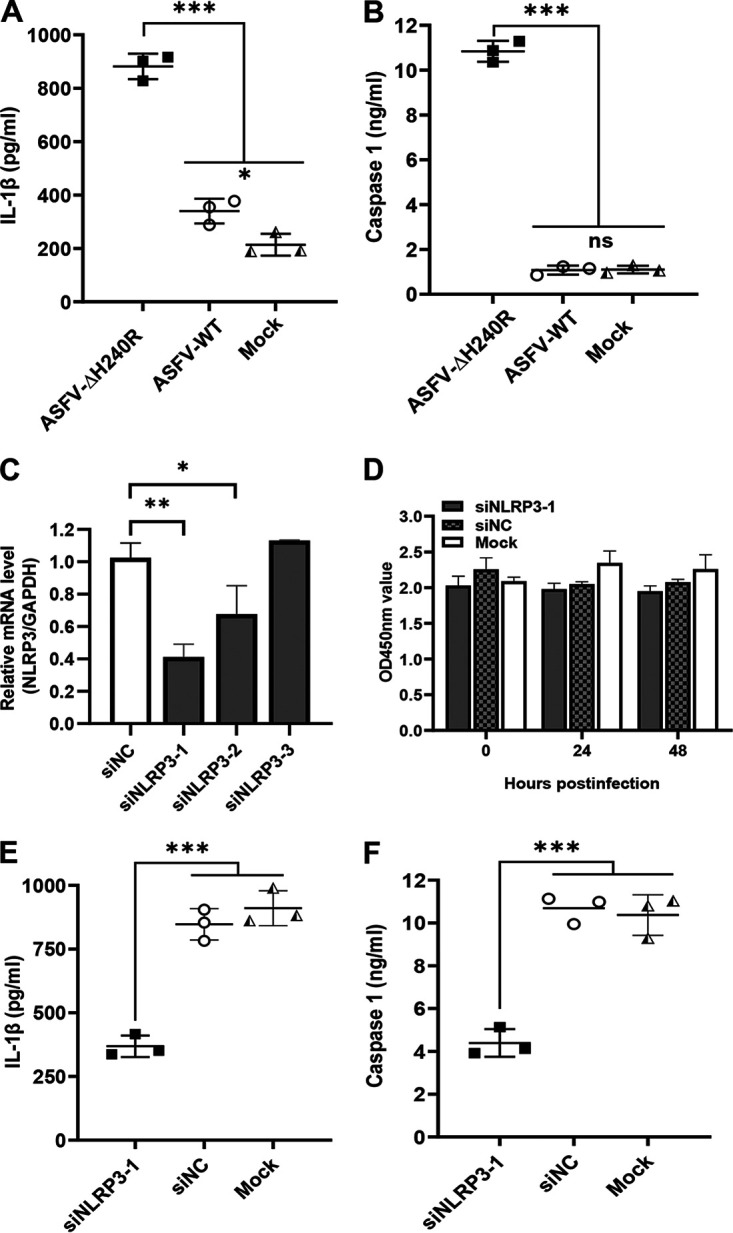
ASFV-ΔH240R infection activates the NLRP3 inflammasome to facilitate IL-1β secretion. (A and B) The production of IL-1β or caspase 1 was higher in PAMs infected with ASFV-ΔH240R than ASFV-WT. PAMs were infected with equal genome copies of ASFV-ΔH240R and ASFV-WT for 24 h. The level of IL-1β in cell supernatants or caspase 1 in cell lysates was measured by ELISA. (C) Validation of knockdown efficiency of siNLRP3 in PAMs. PAMs were transfected with three different siRNAs targeting *NLRP3* (siNLRP3-1, siNLRP3-2, and siNLRP3-3), and the mRNA levels of NLRP3 were measured by RT-qPCR. (D) Cell viability determined with a CCK-8 counting kit. (E and F) The production of IL-1β or caspase 1 in PAMs with NLRP3 knockdown was decreased compared with that in wild-type PAMs upon ASFV-ΔH240R infection. PAMs were transfected with siNLRP3-1 for 24 h and infected with equal genome copies of ASFV-ΔH240R and ASFV-WT for another 24 h. The level of IL-1β in cell supernatants or caspase 1 in cell lysates was measured by ELISA. Data are from three independent experiments. The error bars denote standard errors of the means. The significance of the differences between groups (*n *= 3) was determined using Student’s *t* test (*, *P < *0.05; **, *P < *0.01; ***, *P < *0.001; ns, not significant).

The inflammasome plays a key role in host innate immune responses by promoting procaspase 1 cleavage to generate the activated subunits p20 and p10, ultimately leading to the maturation and secretion of IL-1β. To determine whether ASFV-ΔH240R infection activates the inflammasome, the protein levels of IL-1β and caspase 1 in the PAMs infected with ASFV-ΔH240R or ASFV-WT were measured. As shown by enzyme-linked immunosorbent assay (ELISA), the concentrations of IL-1β in the cell supernatants and the levels of caspase 1 in the cell lysates of the ASFV-ΔH240R-infected PAMs were increased by 3.0-fold (Fig. 6A) and 10.0-fold (Fig. 6B), respectively, compared with those of the ASFV-WT-infected PAMs. Collectively, ASFV-ΔH240R infection induced the activation of caspase 1 and the release of IL-1β from the ASFV-ΔH240R-infected PAMs, suggesting that the inflammasome is activated upon ASFV-ΔH240R infection.

NLRP3 and AIM2 are known to play important roles in virus-induced inflammasome activation (40), but the AIM2 sensor and AIM2-like proteins are absent in porcine cells. We hypothesize that the ASFV-H240R-induced inflammasome activation may depend on NLRP3. Then, NLRP3 expression in PAMs was knocked down with three different small interfering RNAs (siRNAs) targeting the *NLRP3* gene (siNLRP3), and the *NLRP3* mRNA levels were determined by RT-qPCR. As shown in Fig. 6C, the knockdown efficiency of siNLRP3-1 was the most significant. Transfection of the three siRNAs at the tested concentrations resulted in no notable cytotoxicity at various time points (Fig. 6D). To further confirm whether the inflammasome activation induced by ASFV-ΔH240R infection depends on NLRP3, the levels of IL-1β and caspase 1 in wild-type PAMs or the PAMs with NLRP3 knockdown infected with ASFV-ΔH240R or ASFV-WT were measured. As shown in Fig. 6E and F, the concentrations of IL-1β in the cell supernatants and the protein levels of caspase 1 in the cell lysates of the ASFV-ΔH240R-infected PAMs with *NLRP3* knockdown were decreased by 2.5- and 3.1-fold, respectively, compared with those of the ASFV-ΔH240R-infected PAMs with nontargeting siRNA (siNC) transfection. However, there were no significant differences in the protein levels of IL-1β and caspase 1 between the siNC- and siNLRP3-treated PAMs infected with ASFV-WT. The data show that IL-1β maturation as well as caspase 1 cleavage is abrogated in the *NLRP3* knockdown PAMs, suggesting that inflammasome activation induced by ASFV-H240R infection depends on NLRP3.

Taken together, these results show that ASFV-ΔH240R infection specifically activates the NLRP3 inflammasome to promote the secretion of IL-1β.

### pH240R inhibits IL-1β maturation.

**FIG 7:**
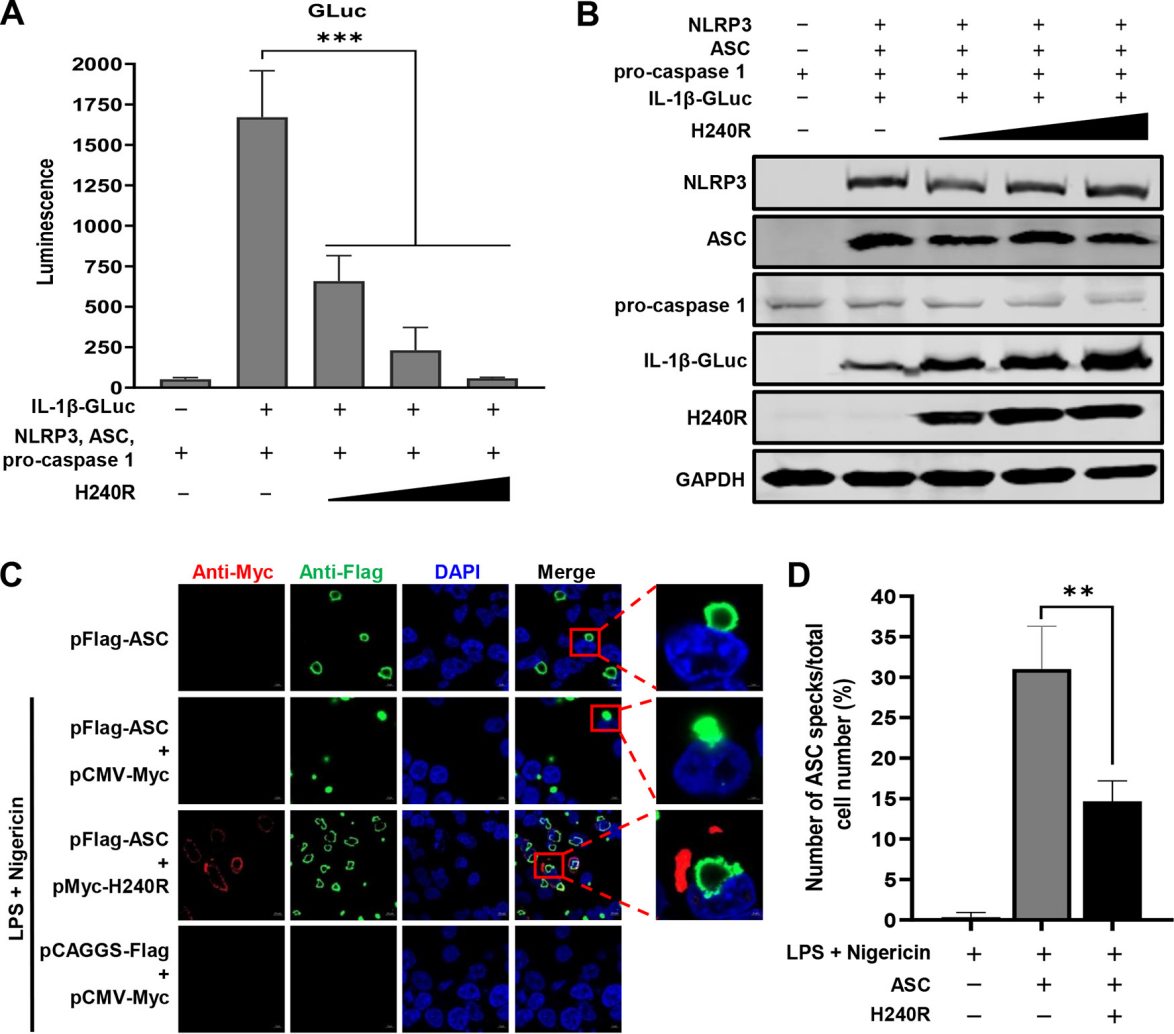
The H240R protein (pH240R) inhibits IL-1β maturation. (A) The inhibitory effect of pH240R on IL-1β secretion was analyzed in the NLRP3 inflammasome reconstitution system. HEK293T cells were cotransfected with the GLuc-based NLRP3 inflammasome system in combination with increasing doses of pH240R, and the supernatants were collected for determination of luciferase activity at 24 h. (B) Expression of the indicated proteins in panel A was analyzed by Western blotting. (C) pH240R inhibited the formation of ASC specks induced by nigericin and lipopolysaccharides. HEK293T cells were cotransfected with pFlag-ASC and pMyc-H240R or pCMV-Myc for 24 h and then treated with nigericin and lipopolysaccharide for another 8 h. Then, the cells were examined by laser confocal microscopy. Bars, 10 μm. (D) Percentages of cells with ASC specks in the experiments whose results are shown in panel C. Data are from three independent experiments. The error bars denote standard errors of the means. The significance of the difference between groups (*n *= 3) was measured using Student’s *t* test (**, *P < *0.01; ***, *P < *0.001).

To evaluate whether pH240R inhibits NLRP3-dependent inflammasome activation, the effects of pH240R on NLRP3 inflammasome activation in HEK293T cells with a *Gaussia* luciferase (GLuc)-based inflammasome reconstruction assay were investigated, and the luciferase activities were measured. As shown in Fig. 7A, transfection with the H240R-expressing plasmid significantly inhibited the NLRP3-dependent GLuc activation in a dose-dependent manner. Ectopic expression of pH240R significantly inhibited IL-1β maturation, but not NLRP3, ASC, or procaspase 1 expression, as demonstrated by NLRP3 inflammasome reconstitution assay (Fig. 7B). These results indicate that pH240R antagonizes the NLRP3-dependent inflammasome activation to inhibit IL-1β maturation.

ASC forms speck-like structures, which are known as pyroptosomes, upon inflammasome activation (41). It has been reported that ASC forms specks when it is ectopically expressed in cells treated with nigericin and LPS, and ASC speck formation correlates with IL-1β processing (42). To investigate whether pH240R inhibits ASC speck formation, HEK293T cells were cotransfected with the *ASC*-expressing plasmid together with *H240R* or control, and then the cells were treated with nigericin and LPS and analyzed by laser confocal microscopy. ASC expression in the presence of nigericin and LPS treatment induced speck formation in most ASC-expressing cells; in contrast, pH240R significantly impaired speck formation (Fig. 7C). The results indicate that pH240R inhibits the NLRP3-dependent inflammasome activation prior to or during ASC speck formation.

### pH240R interacts with NLRP3 and inhibits its oligomerization.

**FIG 8:**
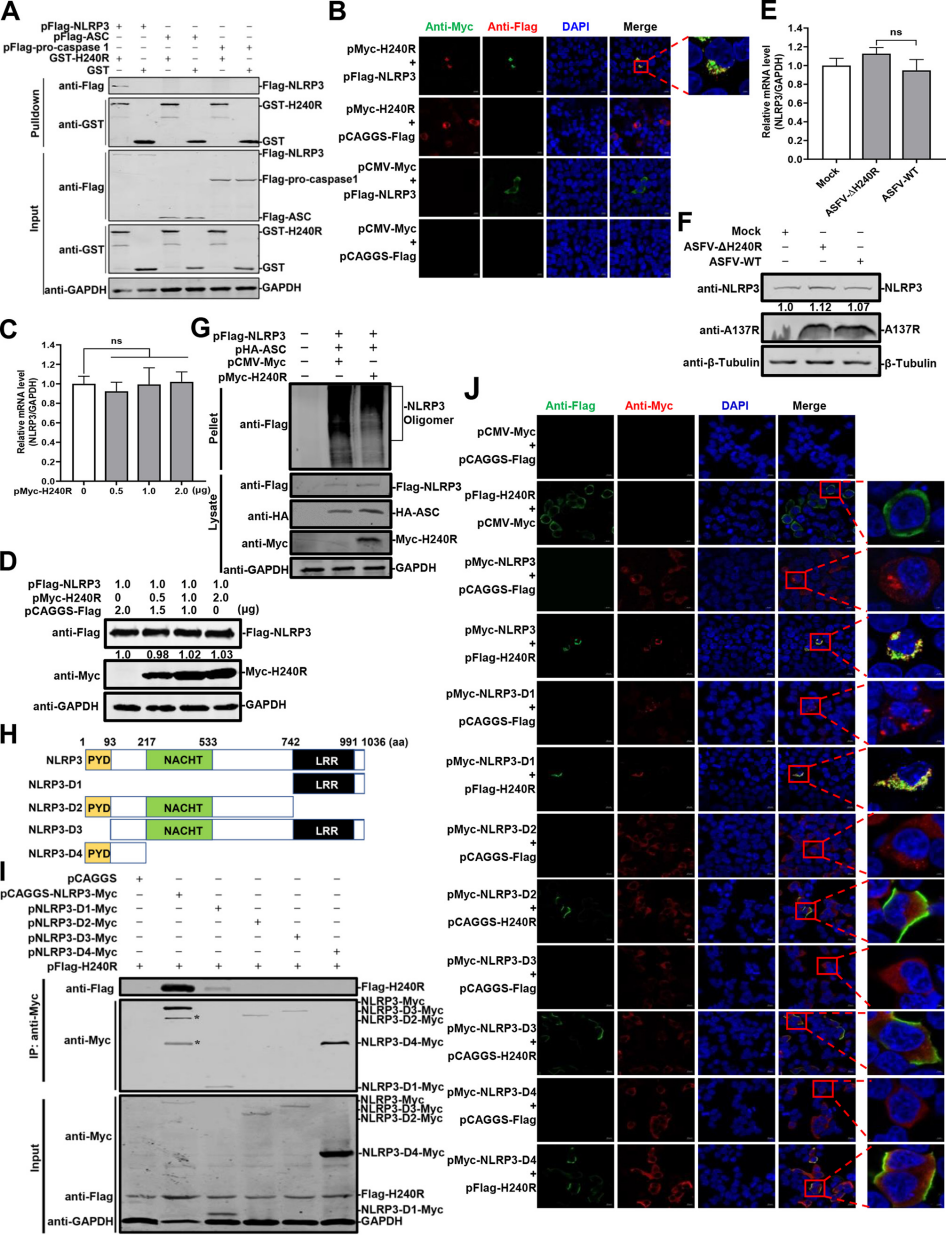
The H240R protein (pH240R) interacts with NLRP3 and inhibits its oligomerization. (A) pH240R interacts with NLRP3. HEK293T cells were transfected with pFlag-H240R. GST pulldown and immunoblotting analyses were performed with an anti-GST or anti-Flag MAb. (B) Colocalization of pH240R with NLRP3. HEK293T cells were cotransfected with pFlag-NLRP3 (1.0 μg) alone or together with pMyc-H240R (1.0 μg) and analyzed by laser confocal microscopy for NLRP3 and pH240R. Bars, 10 μm. (C) The mRNA levels of *NLRP3* were not affected by overexpression of pH240R. HEK293T cells were cotransfected with pFlag-NLRP3 (1.0 μg) and increasing doses of pFlag-H240R (0, 0.5, 1.0, and 2.0 μg) into 12-well plates. The mRNA levels of NLRP3 were examined by RT-qPCR. (D) The expression of NLRP3 was not inhibited by overexpressed pH240R. HEK293T cells were cotransfected with pFlag-NLRP3 (1.0 μg) and increasing doses of pFlag-H240R (0, 0.5, 1.0, and 2.0 μg) into 12-well plates. The protein expression was examined by Western blotting. (E and F) Expression levels of NLRP3 are not affected by pH240R in ASFV-infected PAMs. PAMs were infected with ASFV-WT or ASFV-ΔH240R for 24 h. The mRNA levels and the protein expression of NLRP3 were determined by RT-qPCR and Western blotting. (G) The oligomerization of NLRP3 is inhibited by pH240R. HEK293T cells were cotransfected with the *NLRP3*- and *ASC*-expressing plasmid in combination with the empty or *H240R*-expressing plasmid for 36 h, the cells lysates were prepared in NP-40, and the pellets were washed with PBS three times and cross-linked using 4 mM disuccinimidyl suberate (catalog no. 21555; Invitrogen) for 30 min. (H) Schematic representations of the NLRP3 domains. (I) The PRR region of NLRP3 interacts with H240R. HEK293T cells were cotransfected with pFlag-H240R and the plasmids expressing the Myc-tagged full-length or truncated NLRP3 constructs. The cell lysates were subjected to co-IP analysis using an anti-Myc MAb. *, protein bands that could be the cleaved products of full-length NLRP3. (J) The oligomerization of NLRP3-D1 inhibited by pH240R. HEK293T cells were cotransfected with the *NLRP3*-, *NLRP3-D1-*, *NLRP3-D2*-, *NLRP3-D3*-, or *NLRP3-D4*-expressing plasmid in combination with the empty or *H240R*-expressing plasmid, and the oligomerization of the wild-type and the truncated NLRP3 in the transfected cells was analyzed by laser confocal microscopy. Bars, 10 μm. ns, not significant.

To elucidate the mechanism by which pH240R inhibits the NLRP3-dependent inflammasome activation, we clarified the protein of inflammasome component that interacting with pH240R by glutathione *S*-transferase (GST) pulldown assay. As shown in Fig. 8A, the GST-tagged pH240R (GST-pH240R) protein pulled down the Flag-tagged NLRP3 (Flag-NLRP3), but not Flag-ASC or Flag-procaspase 1. Consistent with the results, colocalization of pH240R and NLRP3 was clearly observed in the cytoplasm with a Pearson’s correlation coefficient of 0.89 (Fig. 8B). Together, these results suggest that pH240R interacts with the NLRP3 protein. Moreover, to investigate whether pH240R affects the transcription or expression of NLRP3, pFlag-NLRP3 and increasing amounts of pMyc-H240R were cotransfected into HEK293T cells. The results showed that neither the transcription nor the translation of NLRP3 was affected by pH240R (Fig. 8C and D). The expression levels of endogenous NLRP3 were not affected by pH240R during ASFV or ASFV-ΔH240R infection (Fig. 8E and F).

We have shown that pH240R inhibits the NLRP3-dependent inflammasome activation prior to or during ASC speck formation; thus, we hypothesized that pH240R may inhibit NLRP3 oligomerization and/or ASC recruitment to NLRP3 oligomers. To determine whether pH240R inhibits the oligomerization of NLRP3, HEK293T cells were cotransfected with the plasmids pFlag-NLRP3, pHA-ASC, and pMyc-H240R to analyze oligomerization as described previously (34). As shown in Fig. 8G, the oligomerization levels of NLRP3 were inhibited by pH240R. Next, HEK293T cells were cotransfected with the plasmids harboring four truncated mutants of *NLRP3* (Fig. 8H), and the *H240R-*expressing plasmid to analyze the NLRP3 domains responsible for the interaction with pH240R by co-IP assay. The results showed that pH240R was coprecipitated with the Myc-tagged NLRP3-D1(743-1036) (Fig. 8I). As shown in Fig. 8J, most of the transfected cells displayed oligomers in the HEK293T cells cotransfected with the NLRP3-D1(743-1036)-expressing plasmid in combination with an empty expression plasmid, while the ratio of the oligomer forms was decreased approximately 40% in the HEK293T cells cotransfected with the NLRP3-D1(743-1036)-expressing plasmid combined with the H240R-expressing plasmid. However, no oligomerization occurred in the cells expressing NLRP3-D2, NLRP3-D3, or NLRP3-D4, and these proteins were dispersed throughout the cytoplasm of the transfected cells.

Taken together, these results indicate that pH240R interacts with NLRP3 and inhibits its oligomerization, thereby inhibiting NLRP3 inflammasome activation.

### Inhibition of NF-κB signaling and NLRP3 inflammasome activation promotes ASFV-ΔH240R replication.

In response to viral infection, macrophages secrete large amounts of cytokines, including IL-1β, to inhibit the replication of viruses. However, the production of many proinflammatory cytokines in response to viral infection is regulated principally at the transcriptional or translational level. Our results described above showed that the viral growth of ASFV-ΔH240R was significantly decreased in PAMs and that NF-κB signaling and NLRP3 inflammasome activation were induced upon ASFV-ΔH240R infection (Fig. 1 and 6).

**FIG 9:**
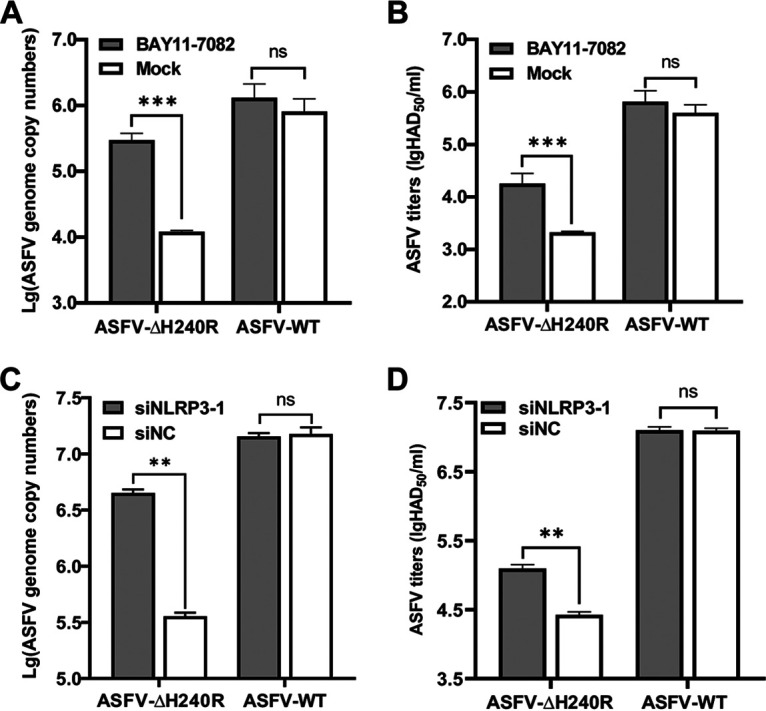
Inhibition of the NF-κB signaling or NLRP3 inflammasome activation promotes ASFV-ΔH240R replication in PAMs. (A and B) Inhibition of the NF-κB signaling promotes ASFV-ΔH240R replication in PAMs. PAMs (10^5^) seeded in 48-well plates were cultured for 12 h, treated with 5 nM BAY11-7082 (an inhibitor of the NK-κB signaling pathway) in RPMI 1640 medium for another 2 h, and then infected with 10^5^ genome copies of ASFV-ΔH240R or ASFV-WT. At 1.5 hpi, the medium was replaced with fresh medium. At 72 hpi, the viral genome copies (A) and viral titers (B) in PAMs infected with ASFV-ΔH240R or ASFV-WT were determined. (C and D) Inhibition of the NLRP3 inflammasome activation promotes ASFV-ΔH240R replication in PAMs. PAMs (10^5^) were transfected with siNLRP3-1 for 24 h, followed by infection with 10^5^ genome copies of ASFV-ΔH240R or ASFV-WT for 72 h, and the viral genome copies (C) and viral titers (D) in the PAMs were quantitated. Data represent three independent experiments. The error bars denote standard errors of the means. The significance of the difference between the groups (*n *= 3) was measured using Student’s *t* test (**, *P < *0.01; ***, *P < *0.001; ns, not significant).

To investigate the effects of NF-κB activation on the replication of ASFV-ΔH240R in PAMs, BAY11-7082, an inhibitor of the NF-κB signaling pathway, was added to PAMs, and the viral genome copies and viral titers in the PAMs infected with ASFV-ΔH240R or ASFV-WT were determined. As shown in Fig. 9A and B, the viral genome copies and the viral titers of ASFV-ΔH240R in the inhibitor-treated PAMs were increased by 25.1- and 9.1-fold, respectively, compared with those in the mock-treated PAMs (*P < *0.01), but there were no significant differences between the medium- and inhibitor-treated PAMs upon ASFV-WT infection, indicating that inhibition of the NF-κB signaling significantly promotes the replication of ASFV-ΔH240R in PAMs.

To further investigate the effects of NLRP3 knockdown on the replication of ASFV-ΔH240R in PAMs, the viral genome copies and viral titers were measured in the siNLRP3- or siNC-transfected PAMs infected with ASFV-ΔH240R or ASFV-WT. The results showed that the viral genome copies and the viral titers of ASFV-ΔH240R in *NLRP3* knockdown PAMs were significantly increased by 10.9- and 4.7-fold, respectively, compared with those in the siNC-treated PAMs, but there were no significant differences between the siNC- and siNLRP3-transfected PAMs (Fig. 9C and D), indicating that inhibition of NLRP3 inflammasome activation significantly promotes the replication of ASFV-ΔH240R in PAMs.

Collectively, the data show that the activated NF-κB signaling and NLRP3 inflammasome induced by ASFV-H240R infection exhibit the antiviral effect.

## DISCUSSION

In the coevolution of hosts and pathogens, natural selection results in a delicate balance between a host’s ability to defend against pathogens and a pathogen’s ability to evade host defenses (43). Previous studies have shown that the secretion of IL-1β, which is regulated by inflammasome activation, is a critical step in the host innate immune responses to infections with various pathogens (9, 10). Upregulated expression of adhesion molecules of endothelial cells targets the activated immune cells to sites of infection via proinflammatory cytokines (9). Moreover, proinflammatory cytokines also activate the NF-κB, JNK, and p38 pathways to upregulate the expression of multiple genes that contribute to the innate immune responses against various pathogens at the sites of infections (44). Components of various pathogens can be recognized by inflammasome sensor proteins, which induce inflammasome activation and proinflammatory cytokine secretion (45, 46). In addition, it has been shown that administration or depletion of proinflammatory cytokines can increase or decrease the load and/or pathogenicity of various pathogens *in vivo*, respectively (44). The production of IL-1β is regulated by two pathways: the transcription of pro-IL-1β mRNA is regulated by NF-κB, which also regulates the synthesis of mRNAs encoding caspase 1 and pro-IL-1β (14–16), and the processing of IL-1β is modulated by the PRR-induced assembly of the inflammasome and the maturation of caspase 1, which leads to the cleavage of pro-IL-1β into the mature IL-1β (17). In this study, a novel mechanism by which ASFV pH240R suppresses NF-κB signaling and NLRP3 inflammasome activation to inhibit IL-1β transcription and maturation was revealed.

The low-level production of IL-1β induced by ASFV infection was identified by us and others (8, 34), indicating that ASFV proteins have evolved multiple strategies to escape from the host immune responses and further suppress IL-1β production. pA238L, which is encoded by the ASFV *A238L* gene, is an IκB homolog protein that significantly inhibits NF-κB activation, thereby inhibiting the expression of IL-1β, but the virulence of ASFV with *A238L* deleted is not attenuated *in vivo* (33). pL83L, which is encoded by the ASFV *L83L* gene, interacts directly with IL-1β, but the virulence of ASFV with *L83L* deleted is not attenuated *in vivo* (32). However, pDP96R, which is encoded by the ASFV *DP96R* gene, inhibits the NF-κB promoter activation by interacting with IKKβ, and the virulence of the *DP96R* deletion mutant is attenuated *in vivo* (47, 48). pMGF505-7R, encoded by the ASFV *MGF505-7R* gene, binds to the IKK complex to inhibit *pro-IL-1β* transcription and interacts with NLRP3 to inhibit IL-1β secretion, and the virulence of ASFV with *MGF505-7R* deleted is attenuated *in vivo* (34).

Our previous study showed that pH240R is a capsid protein that interacts with p72 and is associated with ASFV morphogenesis during the ASFV life cycle (8). We found that ASFV-ΔH240R failed to efficiently assemble after entry into the cells and generated 98% aberrant tubular and bilobulate structures, resulting in major growth defects. In addition, ASFV-ΔH240R infection induced higher expression of proinflammatory cytokines, including IL-1β, IL-6, TNF-α, and C-X-C motif chemokine ligand 8, in PAMs than did ASFV-WT infection (8). In this study, we confirmed that the phosphorylation of IκBα and p65 and the transcription of *pro-IL-1β* were more highly induced by ASFV-ΔH240R infection than ASFV-WT (Fig. 1 and 6), indicating that NF-κB signaling and inflammasome activation were induced in the ASFV-ΔH240R-infected PAMs. To further investigate the molecular mechanisms underlying the pH240R-mediated inhibition of IL-1β production, an NF-κB luciferase reporter and NLRP3 inflammasome reconstruction system were constructed based on the NF-κB and GLuc reporter systems to evaluate the activation of the NF-κB promoter and the production of IL-1β. We further confirmed that pH240R significantly inhibited NF-κB activation (Fig. 2) and IL-1β maturation (Fig. 7).

The inhibition of the IL-1β-mediated inflammatory response associated with various mechanisms, including the first signaling (transcription of pro-IL-1β) and the second signaling (maturation and release of IL-1β) (14–17). Our results showed that pH240R inhibited both the transcription of pro-IL-1β and maturation of IL-1β, indicating that pH240R functions as a pleiotropic regulator of antiviral innate immune responses, including the NF-κB and NLRP3 inflammasome signaling cascades. Once NF-κB signaling is activated, the NF-κB dimer p50/p65 is translocated to the nucleus and induces the expression of proinflammatory cytokines. Viruses have evolved several strategies to inhibit the activation of NF-κB signaling, mainly regulating receptors, the IKK complex, IκBα, or p50/p65. It has been shown that herpes simplex virus 1 ubiquitin-specific protease (UL36USP) inhibits NF-κB activation by deubiquitinating IκBα and restricting its degradation (49). Kaposi’s sarcoma-associated herpesvirus latency-associated nuclear antigen 1 inhibits NF-κB activation by degrading p65 through the ubiquitin-proteasome pathway (50). The human cytomegalovirus UL26 protein inhibits the phosphorylation of IKKβ to antagonize the NF-κB activation (51). ORFV073 inhibits the activation of the IKK complex by interacting with the NEMO protein (52). Herpes simplex virus 1 ICP0 inhibits NF-κB activation by binding to the p65/p50 complex and degrading p50 through the ubiquitin-proteasome pathway (53). In this study, we found that pH240R inhibited NEMO expression by interacting with NEMO to inhibit NF-κB activation, thereby inhibiting the transcription of *pro-IL-1β*. However, effects of pH240R on the functions of other molecules cannot be ruled out.

**FIG 10:**
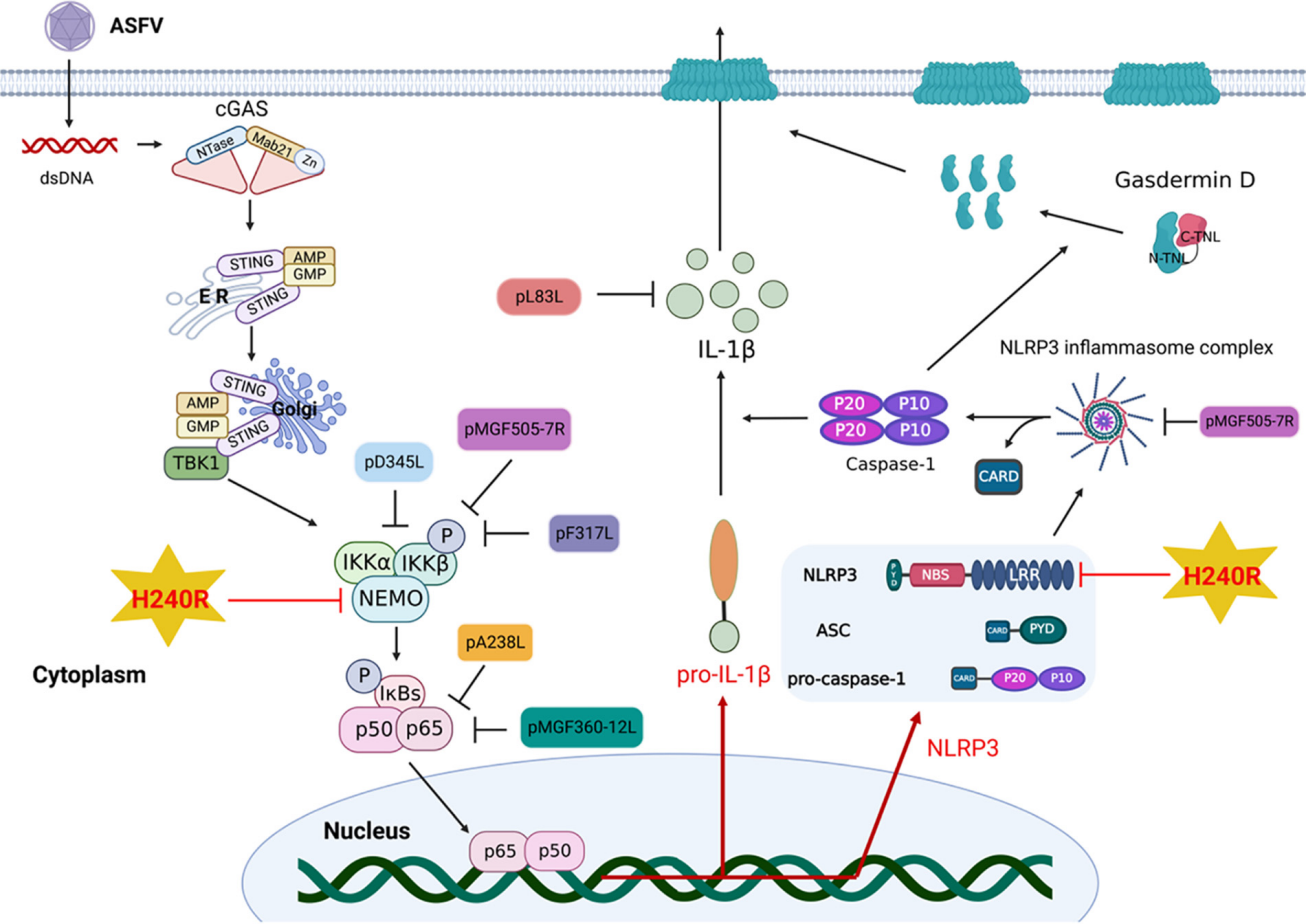
Schematic model of IL-1β maturation inhibited by the ASFV pH240R. Upon ASFV infection, pH240R inhibits IL-1β maturation by antagonizing the activation of NF-κB and inflammasome. pH240R interacts with NEMO to inhibit NF-κB activation and with NLRP3 to inhibit its oligomerization, leading to decreased IL-1β maturation.

When the NLRP3 inflammasome is activated, virus-encoded proteins inhibit IL-1β secretion by disrupting the NLRP3 oligomerization, ASC oligomerization, inflammasome complex formation, caspase 1 activity, and IL-1β maturation. It has been shown that the NS1 protein from influenza A virus inhibits IL-1β maturation by inhibiting caspase 1 activation (54). Overexpression of the influenza A virus NS1 protein in THP-1 cells inhibits NLRP3 inflammasome activation (55). The Sendai virus V protein inhibits ASC oligomerization and NLRP3 self-oligomerization by interacting with NLRP3, thereby inhibiting the secretion of IL-1β (29). In this study, we demonstrated that pH240R interacts with NLRP3 to inhibit its oligomerization, thereby inhibiting IL-1β maturation. Previous studies have shown that several proteins of ASFV, including F317L, MGF505-7R, MGF360-12L, D345L, and I215L, antagonize NF-κB signaling via multiple adaptors of signaling (34, 56–59). We showed here that ASFV-ΔH240R infection induced a strong mature IL-1β response. The possible explanation is that pH240R blocks both the first and second signaling pathways of IL-1β maturation (Fig. 10).

ASFV targets the mononuclear-phagocytic system, and the infected tissues suffer extensive damage following infection with highly virulent isolates (60). ASFV isolates with low virulence also infect these cells, but the degree of tissue involvement and the resulting tissue damage are much less severe (61, 62). ASFV is able to replicate efficiently and induce apoptosis in macrophages *in vivo*, which is likely to be critical to ASFV virulence (60). Although the pathogenicity of ASFV-ΔH240R has not been studied *in vivo*, the biological characteristics of ASFV-ΔH240R infection *in vitro* indicate the possibility of attenuated virulence *in vivo*. On one hand, a viral growth defect would result in a decrease in infection and reduced lymphoid tissue damage, thus permitting a protective immune response to be mounted by the host. On the other hand, ASFV-ΔH240R infection induced high levels of IL-1β production in PAMs. ASFV-ΔMGF505-7R induced the production of high levels of IL-1β in PAMs and pigs and exhibited reduced virulence *in vivo* (34), indicating that cytokine production in pigs is also one of the mechanisms underlying virulence attenuation. However, the virulence of ASFV-ΔH240R in pigs remains to be further investigated.

In summary, we demonstrate for the first time that pH240R inhibits IL-1β production by inhibiting NEMO expression and NLRP3 oligomerization. Our findings provide a novel target for the development of vaccines and drugs for the prevention and control of ASF.

## MATERIALS AND METHODS

### Cells, viruses, and antibodies.

HEK293T cells and PAMs, which were stored in our laboratory, were cultured in RPMI 1640 medium (catalog no. C11875500BT; Gibco) supplemented with 5% antibiotics-antimycotics (10,000 IU/mL penicillin, 10,000 μg/mL streptomycin, and 25 μg/mL amphotericin B) (catalog no. 15240-062; Gibco) and 10% heat-inactivated fetal bovine serum (FBS) (catalog no. 10099-141C; Gibco) in a 37°C incubator in 5% CO_2_. The ASFV pig/Heilongjiang/2018 (ASFV HLJ/2018) strain was isolated from field samples from China as described previously (63). ASFV-ΔH240R was generated in our previous study (8).

A rabbit anti-Myc PAb (catalog no. ab9106; Sigma-Aldrich), a mouse anti-Flag MAb (catalog no. ab62928; Sigma-Aldrich), and a rabbit anti-Flag PAb (catalog no. ab1162; Sigma-Aldrich) were purchased from Sigma-Aldrich. Alexa Fluor 488-conjugated goat anti-rabbit IgG (H+L) (catalog no. 2072687; Invitrogen), Alexa Fluor 488-conjugated goat anti-mouse IgG (H+L) (catalog no. 1942237; Invitrogen), Alexa Fluor 594-conjugated goat anti-rabbit IgG (H+L) (catalog no. 111-585-003; Invitrogen) and Alexa Fluor 568-conjugated goat anti-mouse IgG (H+L) (catalog no. 175697; Invitrogen) antibodies were purchased from Invitrogen. A mouse anti-GST MAb (catalog no. K200006M; Solarbio) and 4′,6-diamidino-2-phenylindole (DAPI) (catalog no. C006; Solarbio) were purchased from Solarbio.

### Construction of plasmids and transfection of cells.

The pMyc-H240R, pFlag-H240R, pGST-H240R, pCAGGS-Flag, pCMV-Myc, and pCAGGS-HA plasmids were described as previously (8). The pFlag-cGAS, pFlag-STING, and pFlag-TBK1 plasmids were described previously (62). The porcine *NLRP3* and *ASC* genes were cloned into the pCAGGS-Flag vector to generate the pFlag-NLRP3 and pFlag-ASC plasmids. Four truncated mutants of the porcine *NLRP3* gene, *NLRP3-D1* (743 to 1036), the amino acids of this domain are retained. *NLRP3-D2* (1 to 742), *NLRP3-D3* (94 to 1036), and *NLRP3-D4* (1 to 217), were cloned into the pCAGGS vector to generate the pNLRP3-Myc, pNLRP3-D1-Myc, pNLRP3-D2-Myc, pNLRP3-D3-Myc, and pNLRP3-D4-Myc plasmids. The porcine IκBα gene was cloned into the pEGFP-C1 vector (Clontech) to generate the pEGFP-IκBα plasmid. The porcine *IKKα*, *IKKβ*, and *NEMO* genes were cloned into the pCMV-Myc vector (Clontech) to generate the pMyc-IKKα, pMyc-IKKβ, and pMyc-NEMO plasmids. The primers used to amplify these genes in this study are listed in Table 1.

**TABLE 1:** Primers used in this study^*a*^

Primer	Sequence (5′–3′)	Description
Flag-NLRP3-F	ATTTTGGCAAAGAATTCATGAGCATGGCAAGCG	For *NLRP3*
Flag-NLRP3-R	CACGATGCGGCCGCTCGAGTTACTTAGACGTTT	
Flag-ASC-F	GAATTCGCCACCATGGGGTGCACGCGTGACG	For *ASC*
Flag-ASC-R	TCACAGATCCTCTTCAGAGATGAGTTTCTGCTC	
Flag-caspase 1-F	GAATTCATGGCCGATAAGGTGCTGAAGGAGGA	For *caspase 1*
Flag-caspase 1-R	CTCGAGTTAATGTCCTGGGAAGAGATAAAAAGA	
Flag-pro-IL-1β-Gluc-F	GGTACCGCCACCATGGCCATAGTACCTGAA	For *pro-IL-1β/Gluc*
Flag-pro-IL-1β-Gluc-R	CTCGAGTCACTTATCGTCGTCACCACCGGCCC	
EGFP-IκBα-F	GGCAAAGAATTCATGTTCCAGCCCGCAGAGCC	For IκBα
EGFP-IκBα-R	CACGATGCGGCCTCATAACGTCAGGCGCT	
Myc-IKKα-F	CTTATGGCCATGGAGGCCCGAATTCGG	For *IKKα*
Myc-IKKα-R	GCGGTACCTCGAGTCATCCTGTTAACCAACT	
Myc-IKKβ-F	TGGAGGCCCGAATTCGGATGAGCTGGTCACCT	For *IKKβ*
Myc-IKKβ-R	GCGGTACCTCGAGTCACGAGGCCTGCTCCAGG	
Myc-NEMO-F	ATGAGCAGGACCCCCTGGAAGAATTCGG	For *NEMO*
Myc-NEMO-R	GCGGTACCTCGAGCTACTCGATACACTCCAT	
NLRP3-D1-Myc-F	ATGGAGGCCCGAATTCGGATGAGCATGGCAAG	For *NLRP3-D1*
NLRP3-D1-Myc-R	CGGCCGCGGTACCTCGAGTTACACAGGCTCAG	
NLRP3-D2-Myc-F	AGCCTGTGTAACTCGAGGTACCGCGGCCGCGG	For *NLRP3-D2*
NLRP3-D2-Myc-R	GCGGCCGCGGTACCTCGAGTTACCAGTTATTG	
NLRP3-D3-Myc-F	ATGGAGGCCCGAATTCGGATGTGGGACAATGC	For *NLRP3-D3*
NLRP3-D3-Myc-R	GCGGCCGCGGTACCTCGAGTTACTGGGAAGGC	
NLRP3-D4-Myc-F	ATGGAGGCCCGAATTCGGATGGAAAAGGAGGA	For *NLRP3-D4*
NLRP3-D4-Myc-R	CGGCCGCGGTACCTCGAGTTACTGGGAAGGC	
IKKα-F	CAATGGAATATTGTTAC	RT-qPCR for IKKα
IKKα-R	GGATTCGATATTTGCAT	
IKKβ-F	GCCAGGGCGGGGATCTGC	RT-qPCR for IKKβ
IKKβ-R	TCTGTTTTCGTGAAGGTA	
NEMO-F	GTTCTGCGAGGCCAGGCG	RT-qPCR for NEMO
NEMO-R	TGCTCCTTGTCCTCTGCCA	
IκBα-F	TGCACTTGGCCATCATCCA	RT-qPCR for IκBα
IκBα-R	GCTCAGGATCACAGCCA	
NLRP3-F	GTACCTTTCCAGAATCTCTA	RT-qPCR for NLRP3
NLRP3-R	GTTCACACTCTCACCCA	
TNF-α-F	CCTACTGCACTTCGAGGTTATC	RT-qPCR for TNF-α
TNF-α-R	ACGGGCTTATCTGAGGTTTG	
IL-1β-F	ACCCAAAACCTGGACCTTGG	RT-qPCR for IL-1β
IL-1β-R	CATCACAGAAGGCCTGGGAG	
IL-6-F	CTCATTAAGTACATCCTCGG	RT-qPCR for IL-6
IL-6-R	GTCTCCTGATTGAACCCAGA	
GAPDH-F	GAAGGTCGGAGTGAACGGATTT	RT-qPCR for GAPDH
GAPDH-R	TGGGTGGAATCATACTGGAACA	

a F, forward; R, reverse.

HEK293T cells were transfected with plasmids (2 μg each) using 2 μL of X-tremeGENE HP DNA transfection reagent (catalog no. 06366236001; Roche) in 6-well plates according to the manufacturer’s instructions. The cells were incubated for 48 h.

### RNA extraction and RT-qPCR.

Total RNA was extracted from HEK293T cells or PAMs using RNAiso Plus (catalog no. 9109; TaKaRa) according to the manufacturer’s protocols. The RNA was treated with DNase (catalog no. 143582; Roche) to remove genomic DNA. The cDNA was processed in a 20-μL volume with avian myeloblastosis virus (AMV) reverse transcriptase XL (catalog no. 2621; TaKaRa) and used as a template for RT-qPCR. The *pro-IL-1β*, *IL-6*, *TNF-α*, *IKKα*, *IKKβ*, *NEMO*, *IκBα*, and *NLRP3* cDNA levels were quantified by RT-qPCR in a reaction mixture containing 10 μM *pro-IL-1β*, *IL-6*, *TNF-α*, *IKKα*, *IKKβ*, *NEMO*, *IκBα*, and *NLRP3* forward and reverse primers (Table 1) and 2 μL of the cDNA template under the condition of 30 s at 95°C, followed by 40 cycles of 95°C for 15 s, 60°C for 30 s, and 72°C for 30 s. *GAPDH* was used as the internal control in this experiment.

### Dual-luciferase reporter assay.

HEK293T cells were cotransfected with reporter plasmids (pNF-κB-FLuc or pIRF3-FLuc [0.1 μg]) and pTK-RLuc (0.01 μg) with or without the indicated expression plasmids using X-tremeGENE HP (catalog no. 6366546001; Roche). At 24 h posttransfection (hpt), the cells were washed with phosphate-buffered saline (PBS) three times, 80 μL of passive lysis buffer was added in each well followed by incubation on a shaker for 20 min at 4°C, and then the luciferase activities were measured using a dual-luciferase reporter assay system (catalog no. E1910; Promega) according to the manufacturer’s instructions. The results are presented as the ratios of firefly luciferase (FLuc) to *Renilla* luciferase (RLuc).

### Co-IP and Western blotting assays.

HEK293T cells were cotransfected with the pMyc-H240R and pFlag-cGAS, pFlag-STING, or pFlag-TBK1 (2 μg each), or with pFlag-H240R and pMyc-IKKα, pMyc-IKKβ, or pMyc-NEMO (2 μg each) for the co-IP assay. At 48 hpi, the cells were lysed with NP-40 for 20 min and centrifuged for 12 min at 4°C; the supernatants in loading buffer were boiled for 8 min at 100°C. The supernatants were incubated with the indicated primary antibodies in the presence of protein G-agarose (catalog no. IP10-10ml; Merck-Millipore) at 4°C overnight. The agarose was washed five times with PBS, and the protein expression was analyzed by Western blotting as described previously (62).

### Measurement of inflammatory-cytokine levels.

A total of 4 × 10^6^ PAMs in 6-well plates were infected with 10^7^ genome copies of ASFV-ΔH240R or ASFV-WT for 1.5 h at 37°C and then washed twice with PBS. The PAMs were then cultured in fresh medium for 24 h. The cell supernatants were collected to measure the concentrations of cytokines using ELISA kits (Ray Biotech, Norcross, GA). The cells were collected for RT-qPCR and Western blotting, and uninfected cells were used as a negative control. All the samples were analyzed in triplicate.

### RNA interference assay.

siRNAs targeting the porcine *NLRP3* genes were synthesized by Gemma. The siRNA sequences targeting *NLRP3* were GCU GCA AGC UGG CUC GUU ATT (siNLRP3-1), GCC UGU GCA CAC GGU AGU ATT (siNLRP3-2), and CCU GUU CAC CAU GUG CUU UTT (siNLRP3-3). The siNC sequence was UUC UCC GAA CGU GUC ACG UTT. A total of 5 × 10^5^ PAMs were seeded in 24-well plates and cultured for 12 h. The PAMs were transfected with 100 nM siNLRP3 or siNC using the XtremeGENE siRNA transfection reagent (catalog no. 4476093001; Roche) according to the manufacturer’s instructions. At 24 hpt, the transfected PAMs were infected with ASFV-ΔH240R for 1.5 h, and the supernatants were washed with PBS and replaced with fresh medium. At 72 hpi, the supernatants and cell lysates were harvested to measure the viral titers and genomic copies.

### Cell viability assay.

A cell viability assay was performed with the Cell Counting Kit-8 (CCK-8) (catalog no. CK04; Dojindo) according to the manufacturer’s instructions.

### Reconstitution of the NLRP3 inflammasome.

An NLRP3 inflammasome reconstitution assay was performed as described previously (64). Briefly, HEK293T cells were seeded in a 24-well plate and cotransfected with the pFlag-NLRP3 (10 ng/well), pFlag-ASC (10 ng/well), pFlag-procaspase 1 (12.5 ng/well), and pFlag-Gluc-pro-IL-1β (pFlag-Gluc) (100 ng/well) plasmids, combined with the pCMV-Myc and pCMV-H240R plasmids (100, 250, and 500 ng/well). At 24 hpt, the luciferase activities of pFlag-Gluc in the cell-free supernatants were measured using a Pierce *Gaussia* luciferase flash assay kit (catalog no. 16159; Thermo Scientific) according to the manufacturer’s instructions.

### Laser confocal microscopy.

To analyze the colocalization of pH240R and NEMO, HEK293T cells were cotransfected with pFlag-H240R and pCMV-Myc, pMyc-IKKα, pMyc-IKKβ, or pMyc-NEMO for 24 h. To confirm the colocalization of pH240R and NLRP3, HEK293T cells were cotransfected with pCMV-H240R and pFlag-NLRP3 for 24 h. For the ASC speck formation assay, HEK293T cells were transfected with pMyc-H240R or pCMV-Myc and pFlag-ASC for 24 h and treated with nigericin and LPS for an additional 6 h to confirm the specific formation of specks. Then, the cells were incubated with the relevant antibodies and examined with a Zeiss LSM 880 laser-scanning confocal microscope as described previously (65).

### GST pulldown assay.

GST pulldown was performed as described previously (9). Briefly, the purified GST-H240R or GST protein was incubated with lysates from cells transfected with the pFlag-NLRP3 plasmid at 4°C for 12 h. The amounts of proteins pulled down by glutathione-Sepharose 4B resin (catalog no. 17-0756-01; GE Healthcare) were measured by Western blotting using a rabbit anti-Flag PAb or a mouse anti-GST MAb.

### Virus inhibition assay.

A total of 10^5^ PAMs were seeded in 48-well plates for 12 h. The PAMs were treated with 5 nM BAY11-7082 (catalog no. HY13453; MedChemExpress), an inhibitor of the NK-κB signaling pathway, in RPMI 1640 medium for 2 h. The cells were infected with 10^5^ genome copies of ASFV-ΔH240R or ASFV-WT. After 1.5 h, the supernatants were replaced with fresh medium. At 72 hpi, the cell lysates and supernatants were harvested to measure viral titers and genome copies.

### qPCR.

ASFV genomic DNA was extracted from the cells and cell supernatants using the MagaBio plus virus DNA purification kit (catalog no. 9109; BioFlux) according to the manufacturer’s protocols. ASFV genome copies were determined with the QuantStudio system (Applied Biosystems, USA) by qPCR.

### Statistical analysis.

Statistical analyses were performed using SPSS 22.0 software (SPSS Software Inc.). Differences between groups were examined for statistical significance using Student’s *t* test. An unadjusted *P* value of <0.05 was considered significant.

### Data availability.

The sequencing data the ASFV HLJ/2018 strain have been deposited at the National Center for Biotechnology Information under GenBank accession no. MK333180.1.
